# Exploiting the mTOR paradox for disease prevention

**DOI:** 10.18632/oncotarget.712

**Published:** 2012-10-17

**Authors:** Ramiro Iglesias-Bartolome, J. Silvio Gutkind

**Affiliations:** Oral and Pharyngeal Cancer Branch, National Institute of Dental and Craniofacial Research, National Institutes of Health, Bethesda, MD, USA; Oral and Pharyngeal Cancer Branch, National Institute of Dental and Craniofacial Research, National Institutes of Health, Bethesda, MD, USA

**Figure 1:**
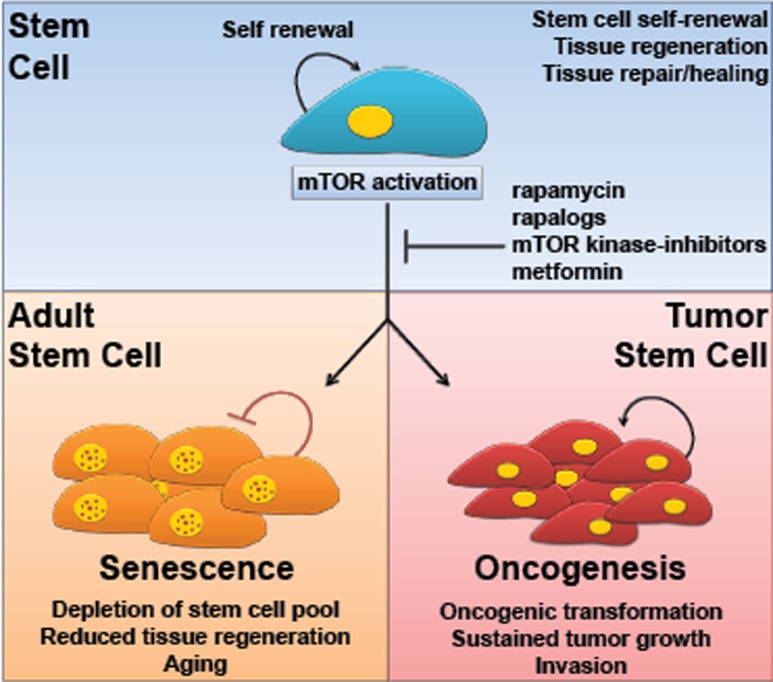
The mTOR paradox mTOR activation contributes to the transformation and growth of cancer cells but the prolonged stimulation of mTOR can also lead to stem cell depletion through the activation of senescence programs. The use of mTOR inhibitors or therapeutic agents resulting in inactivation of mTOR might protect adult stem cells from initiating premature cell senescence programs while concomitantly preventing tumor cell growth.

Activation of the mechanistic target of rapamycin (mTOR) is a common feature in most human malignancies [1]. Inhibition of mTOR by rapamycin and newly developed inhibitors has already shown promising antitumoral activity in a variety of experimental cancer models and in recent clinical trials [2, 3]. However, an increase in mTOR activity has also been related to aging, causing stem cells to undergo differentiation or senescence, thereby exiting the proliferative cell pool ([4-7]; reviewed in [8, 9]). The emerging picture is that while mTOR activation contributes to cancerous growth in transformed cells, the prolonged stimulation of mTOR in normal cells can lead to stem cell depletion and reduced organismal health and life-span. We refer to this effect as the “mTOR paradox” (Figure 1).

Although the molecular mechanism(s) underlying these paradoxical effects of mTOR are not completely understood, this concept is quite relevant in the context of the potential use of mTOR inhibitors for cancer treatment. mTOR inhibitors may prevent the growth of cancer cells that are addicted to, and hence dependent on mTOR function for their aberrant growth, while mTOR inhibition might concomitantly improve the health of normal tissues by protecting their tissue resident stem cells. mTOR drives conversion of reversible cell cycle arrest into irreversible senescence, whereas rapamycin suppresses geroconversion [8]. As part of our laboratory's translational efforts in the area of prevention and treatment of oral malignancies, we have analyzed the benefits of combining rapamycin with radiation, one of the most frequently used therapeutic options for patients with oral cancer [5]. While rapamycin did not significantly increase the anti-cancer effectiveness of radiation when combined, at least in cell culture studies, we made a quite surprising observation. As a control for these experiments, we used normal epithelial cells that we have isolated and grown from the gingiva of normal healthy human volunteers. We found that when the oral keratinocytes, which include epithelial stem cells, were treated with rapamycin and then irradiated, these cells were protected from the overall deleterious effect of radiation on cell growth. Further analysis revealed that mTOR inhibition protects the epithelial stem cells for undergoing senescence by reducing oxidative stress. Senescence resembles cell aging as it renders stem cells unable to grow and repair damaged tissues. In this case, by the simple pre-treatment with rapamycin, we were able to prevent the depletion of tissue regenerating stem cells after radiation. We then applied this finding to an *in vivo* situation in a mouse model, and found that rapamycin protected the oral mucosa from radiation-induced tissue damage, similar to what we observed in human cells in culture.

Radiation therapy is one of the most widely used cancer treatments [10]. In patients with oral cancer, radiation of the head and neck area can result in a side effect called mucositis, a debilitating condition involving painful and deep ulcerations on the oral cavity as a result of damage to the normal tissue. Mucositis causes distress to the patients and results also in substantial increase in patient care cost [11]. In our study, we observed that short term treatment with rapamycin can reduce the undesired effects of radiation in the normal tissues, and prevents the appearance of mucositis in a mouse model. Since rapamycin is an FDA approved drug, this study may provide the basis for further testing in humans. Mucositis prevention would have a remarkable impact in the quality of life and recovery of cancer patients, and at the same time, it would be expected to reduce the treatment cost as it would prevent further complications that need immediate medical assistance. Certainly, the systemic use of mTOR inhibitors may cause multiple undesirable side effects, including the potential impact on the immune system, which will have to be considered with caution. While there are multiple risks associated with the prolonged systemic use of mTOR inhibitors, we can speculate that local mTOR inhibition may have a direct impact in preventing the loss of epithelial stem cells due to genetic or environmental stress conditions, such as those resulting in premature aging.

Rapamycin and other mTOR inhibitors have been shown to prevent cellular senescence in cell culture in all cell types tested [8]. We can then hypothesize that this remarkable effect on stem cell protection can also be potentially applied to other tissues that are persistently exposed to oxidative stress and damage, such as the skin, which is characterized by an age-associated decline in the number and function of its tissue-regenerative stem cells. Indeed, local inhibition of mTOR may prevent premature aging of the skin without the potential risk of increasing cancer incidence. Finally, by exerting distinct effects on cancer and normal cells, mTOR inhibitors may become attractive agents for exploring their use in combination with available anti-cancer therapies.

Overall, we are beginning to understand how molecular circuitries are differentially wired in normal and cancer cells, and how we can perturb distinct signaling pathways to prevent tumor growth without disrupting the function of normal tissues and cells. We expect that additional molecules that play a similar paradoxical distinct role in cancer and normal cells will soon be identified, expanding the tool box of druggable targets for cancer prevention and treatment. These efforts may shed some interesting surprises, as we may be able to find feasible approaches to protect the stem cells residing in each adult tissue from the detrimental impact of environmental assaults that lead to their loss of tissue regenerative capacity and accelerated aging. Ultimately, by protecting adult stem cells from initiating premature cell senescence programs with pharmacological agents that simultaneously prevent tumor cell growth, we may be in the future in a position to delay aging without the potential increase in cancer risk.
